# Electronic gap characterization at mesoscopic scale via scanning probe microscopy under ambient conditions

**DOI:** 10.1038/s41467-022-32439-1

**Published:** 2022-08-08

**Authors:** Dian Li, Xiong Wang, Xiaoyong Mo, Edmund C. M. Tse, Xiaodong Cui

**Affiliations:** 1 Department of Physics, Guangdong-Hong Kong Joint laboratory of Quantum Matter, University of Hong Kong, Pokfulam Road, Hong Kong SAR, P. R. China; 2 Department of Physics, Shantou University, Shantou, 515063 Guangdong P. R. China; 3 Department of Chemistry, HKU-CAS Joint Laboratory on New Materials, University of Hong Kong, Pokfulam Road, Hong Kong SAR, P. R. China

**Keywords:** Characterization and analytical techniques, Characterization and analytical techniques, Characterization and analytical techniques

## Abstract

Electronic gaps play an important role in the electric and optical properties of materials. Although various experimental techniques, such as scanning tunnelling spectroscopy and optical or photoemission spectroscopy, are normally used to perform electronic band structure characterizations, it is still challenging to measure the electronic gap at the nanoscale under ambient conditions. Here we report a scanning probe microscopic technique to characterize the electronic gap with nanometre resolution at room temperature and ambient pressure. The technique probes the electronic gap by monitoring the changes of the local quantum capacitance via the Coulomb force at a mesoscopic scale. We showcase this technique by characterizing several 2D semiconductors and van der Waals heterostructures under ambient conditions.

## Abstract (web summary)

The nanoscale measurement of electronic bandgaps is crucial for material characterization, but it is usually performed under cryogenic conditions. Here, the authors report a scanning probe method to determine the electronic bandgap of 2D semiconductors at the nanoscale by monitoring their quantum capacitance under ambient conditions.

## Introduction

Electronic gaps, either bandgaps for crystals or highest occupied molecular orbital-lowest unoccupied molecular orbital (HOMO-LUMO) gaps for molecular systems play a dominating role in material electric properties. Although there are various experimental techniques, such as optical^1^ and photoemission spectroscopy^2^, electric transports^3^, etc., it is still challenging to realize electronic gap characterization with nanometre spatial resolution. Despite a wide range of functionalities of the scanning probe microscopy (SPM)^4^, the scanning tunnelling spectroscopy (STS)^5–8^, a working mode of scanning tunnelling microscopy (STM)^9^, is probably the only widely used technique capable of probing the electronic gap with a nanometre resolution. Generally, STS provides the differential conductance ${dI}/{dV}$ of the tunnelling current as a function of the fermi level difference between the sample and the sharp metal tip. This differential conductance ${dI}/{dV}$ reflects the electron’s local density of states (LDOS) and therefore the gap state could be extracted. The atomic resolution of STM makes STS a gap characterizing tool with ultimate spatial resolution. However, STS suffers many limitations inherited from STMs. As the tunnelling current depends exponentially on the spatial separation between the STM tip and samples, mechanical vibrations even thermal fluctuation easily blurs the needed information. This hinders the implement of STS only viable at cryogenic temperature.

In this work, we demonstrate a technique of SPM for electronic gap characterization with a nanometre spatial resolution while immune to environmental restrictions. The technique scrutinizes the local electric field as a function of the Fermi level difference between the sample and the conductive atomic force microscopy (AFM) tip and subsequently extracts the electronic gap from the measured local quantum capacitance fluctuation. The nature of the long-range Coulomb field relaxes the strict requirement on environmental stabilities and makes this technique applicable under ambient conditions.

## Results and discussion

### Working principle

**Fig. 1:**
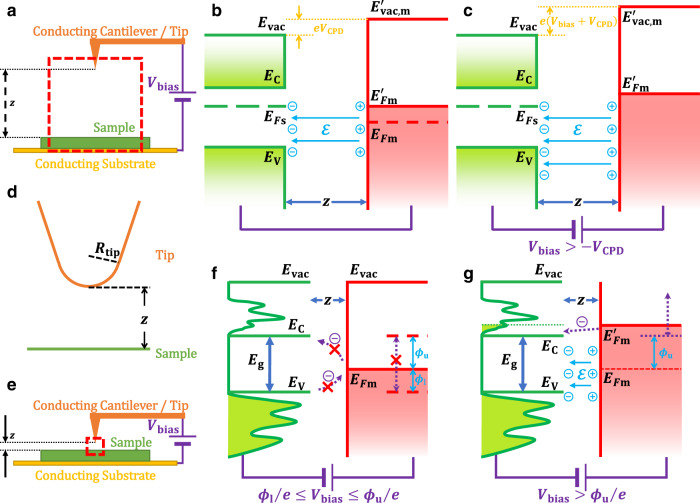
Working principle of localized electric force microscopy (LEFM). **a** The schematic diagram of the regular electric force microscopy (EFM). Here ${V}_{{{{{{\rm{bias}}}}}}}$ is the scanning external bias voltage and $z$ is the tip-sample separation. **b** The energy level alignment between the conductive tip and the sample at the regular EFM without bias (${V}_{{{{{{\rm{bias}}}}}}}=0$). Here ${E}_{{{{{{\rm{vac}}}}}}}$, ${E}_{{{{{{\rm{C}}}}}}}$, ${E}_{F{{{{{\rm{s}}}}}}}$, and ${E}_{{{{{{\rm{V}}}}}}}$ denote the vacuum level, conduction band edge, Fermi level, and valance band edge of the sample, respectively. The charge transfer between the sample and tip aligns the Fermi level ${E}_{F{{{{{\rm{m}}}}}}}$ and consequently twists the Fermi level to ${E}_{F{{{{{\rm{m}}}}}}}^{{\prime} }$ and vacuum level to ${E}_{{{{{{\rm{vac}}}}}},{{{{{\rm{m}}}}}}}^{{\prime} }$ by the amount of contact potential difference ${V}_{{{{{{\rm{CPD}}}}}}}$, and builts up a local electric field ${{{{{\mathcal{E}}}}}}$ between the samples and the tip. **c** The energy alignment between the tip and the sample under an external bias at finite temperature in regular cases. **d** The capacitor model of the EFM system which consists of the conductive AFM tip with an apex curve of ${R}_{{{{{{\rm{tip}}}}}}}$ and the sample plane. **e** The schematic diagram of the LEFM model in which the EFM tip is pushed close to the sample and the local electric field is dominated by a nanoscale area. **f** When the external bias is not large enough to push the tip’s Fermi level out of the energy bandgap ${E}_{{{{{{\rm{g}}}}}}}$, charge transfer cannot take place and ${E}_{F{{{{{\rm{m}}}}}}}$ is pinned (purple arrows blocked by red crosses). Here ${\phi }_{{{{{{\rm{u}}}}}}}$/${\phi }_{{{{{{\rm{l}}}}}}}$ denotes the upper/lower energy difference between ${E}_{F{{{{{\rm{m}}}}}}}$ and ${E}_{{{{{{\rm{C}}}}}}}$/${E}_{{{{{{\rm{V}}}}}}}$. **g** When ${V}_{{{{{{\rm{bias}}}}}}}$ pushes ${E}_{F{{{{{\rm{m}}}}}}}$ in alignment with the conduction or valance band of the sample, the adequate density of states makes charge transfer possible (purple arrows) and electric field ${{{{{\mathcal{E}}}}}}$ is built up consequently.

In regular electric force microscopy (EFM)^10^ charge transfer aligns the Fermi or quasi-Fermi level between samples and the conductive AFM tip, and consequently twists the local surface vacuum levels by a so-called contact potential difference, ${V}_{{{{{{\rm{CPD}}}}}}}$, which reflects the energy difference between the (quasi) Fermi levels of the tip and the sample. This ${V}_{{{{{{\rm{CPD}}}}}}}$ builds a local electric field between the samples and the tip (Fig. 1b). This electric field could be probed with the Coulomb force acting on the conductive AFM tip as sketched in Fig. 1a. Generally, under an external bias voltage ${V}_{{{{{{\rm{bias}}}}}}}$ between the sample and the tip (Fig. 1c), the electrostatic energy ${U}_{{{{{{\rm{el}}}}}}}$ of the entire system could be simply described by a charging capacitor model

1 $${U}_{{{{{{\rm{el}}}}}}}\left(z,\;{V}_{{{{{{\rm{bias}}}}}}}\right)=-\frac{1}{2}{C}_{{{{{{\rm{s}}}}}}}\left(z\right){({V}_{{{{{{\rm{bias}}}}}}}+{V}_{{{{{{\rm{CPD}}}}}}})}^{2}$$

where the capacitance ${C}_{{{{{{\rm{s}}}}}}}$ is the effective capacitance of the entire system consisting of the tip and the sample. In a simple but realistic model^11,12^, the electrostatic capacitance is approximated by

2 $${C}_{{{{{{\rm{s}}}}}}}\left(z\right)=2\pi {\epsilon }_{0}{\epsilon }_{r}{R}_{{{{{{\rm{tip}}}}}}}{{{{{\rm{ln}}}}}}\left(1+\frac{{R}_{{{{{{\rm{tip}}}}}}}}{z}\right)$$

where the capacitor could be modelled by a conductor cone with an apex curve of ${R}_{{{{{{\rm{tip}}}}}}}$ and a semi-infinite plane, separated by $z$ (Fig. 1d). The electrostatic force added to the tip could be described by the negative gradient of ${U}_{{{{{{\rm{el}}}}}}}$, i.e.,

3 $${F}_{{{{{{\rm{el}}}}}}}\left(z,\;V\right)=-\frac{\partial {U}_{{{{{{\rm{el}}}}}}}\left(z\right)}{\partial z}=\frac{1}{2}\frac{\partial {C}_{{{{{{\rm{s}}}}}}}\left(z\right)}{\partial z}{({V}_{{{{{{\rm{bias}}}}}}}+{V}_{{{{{{\rm{CPD}}}}}}})}^{2}$$

**Fig. 2:**
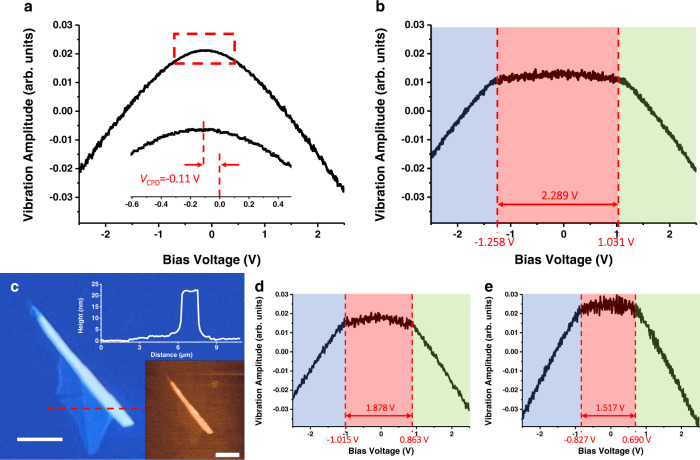
LEFM measurement on MoS_2_ sample. **a** The representative vibration amplitude vs. the external bias curves on monolayer MoS_2_ under the regular EFM working mode. The inset is the zoom-in of the apex area. The offset of the parabolic curve from the zero bias indicates ${V}_{{{{{{\rm{CPD}}}}}}}$. The representative curves of vibration amplitude vs. external bias on **b** monolayer, **d** bilayer, and **e** multilayer (thickness ∼22 nm) MoS_2_ with the LEFM working mode. The bias ranges corresponding to the valance band, band gap, and conduction band of the samples are shaded in blue, red, and green respectively and the band edges are marked by the red dashed lines. **c** The optical image of the few-layer MoS_2_ samples on an ITO substrate. The insets present the corresponding AFM topography mapping and the height profile along the red dashed line (scale bars: 5 μm).

If we scan the external bias, the electrostatic force displays a parabolic curve as sketched in Fig. 2a. The contact potential difference could be extracted easily from the bias voltage corresponding to the minimum of the force *vs*. external bias curve^13^. This is similar to how the scanning Kevin probe microscopy works^14^. If the tip is close enough to the sample where the Coulomb field is dominated by the charges on the local mesoscopic area (Fig. 1e), the quantum states to accommodate free electrons(holes) in the sample’s domain are limited owing to its finite LDOS. Namely, the finite electron states owing to the limited LDOS in a mesoscopic volume/area affect the system capacitance with a form of quantum capacitance *C*_*q*_^15,16^. Starting from the definition of the capacitance we could write the quantum capacitance ${C}_{{{{{{\rm{q}}}}}}}$ of the domain as a function of the local density of states,

4 $${C}_{{{{{{\rm{q}}}}}}}\left(u\right)=\frac{{dQ}\left(u\right)}{{du}}={Ae}\int_{0}^{{{\infty }}}\frac{\partial f\left(V-u\right)}{\partial u}{{{{{\rm{DOS}}}}}}\left(V\right){dV}$$

where $A$ is the area or volume of the domain depending on a 2D or 3D case, *u* is the chemical potential/Fermi level, $e$ is the electron charge, and $f\left(\varepsilon \right)$ is the Fermi-Dirac distribution function, respectively.

Strictly speaking, the system capacitance is a combination of both electrostatic capacitance and the quantum capacitance of the local domain in series, say ${C}_{{{{{{\rm{s}}}}}}}=\frac{{C}_{{{{{{\rm{e}}}}}}}{C}_{{{{{{\rm{q}}}}}}}}{{C}_{{{{{{\rm{e}}}}}}}+{C}_{{{{{{\rm{q}}}}}}}}$. In a rough estimate of 2D materials, if we take ${{{{{\rm{DOS}}}}}}={m}_{{{{{{\rm{e}}}}}}}^{*}/\pi {\hslash }^{2}$ (here the effective mass of the electron ${m}_{{{{{{\rm{e}}}}}}}^{*}\; \approx \; 0.5{m}_{{{{{{\rm{e}}}}}}}$ for monolayer MoS_2_, MoSe_2_, etc.), the quantum capacitance per unit area is around the orders of magnitude of 10^22^ eV^−1^cm^−2^ vs. 10^20^ eV^−1^cm^−2^ of the extreme electrostatic capacitance of a monolayer. Therefore, we usually ignore the contribution of quantum capacitance ${C}_{{{{{{\rm{q}}}}}}}$ as ${C}_{{{{{{\rm{q}}}}}}}\;\gg \;{C}_{{{{{{\rm{e}}}}}}}$ and ${C}_{{{{{{\rm{s}}}}}}}\; \approx \; {C}_{{{{{{\rm{e}}}}}}}$. Nonetheless, the definition of quantum capacitance (Eq. 4) implies that the quantum capacitance in mesoscopic islands decreases as the effective area/volume shrinks and the quantum capacitance drops close to zero at finite temperature when the Fermi level is within the energy gap. If ${C}_{{{{{{\rm{q}}}}}}}$ is comparable to or even smaller than ${C}_{{{{{{\rm{e}}}}}}}$, the quantum capacitance kicks in. Namely, if the external bias pushes the Fermi level within the energy gap of the sample (Fig. 1f), there is no vacancy to accommodate extra charge transfer between the tip and the sample, and consequently, the local electric field is pinned. Theoretically, under this proposed EFM working mode, one can predict that the force vs. external bias curve could reflect the LDOS and consequently reveal the electronic gap of the sample at the local domain. For the sake of distinction, we use LEFM (localized electric force microscopy) to distinguish the proposed EFM working mode from the regular EFM.

### LEFM measurement on TMDC samples

Figure 2a shows a paradigm of the regular EFM working mode on the popular monolayer transition metal dichalcogenide (TMDC) MoS_2_ on indium tin oxide (ITO) glasses. Under the regular EFM mode (dynamic force/non-contact mode under an external bias with a lifted distance from the sample), the vibration amplitude which reflects the force added on the EFM tip displays a parabolic response to the external bias. The offset from the zero bias of the parabolic curve (inset of Fig. 2a) indicates the contact potential difference^17^. As shown in Supplementary Fig. 1, the parabolic response is further enhanced as we reduce the separation between the tip and the sample, which is fully expected by classical electrostatics (Eq. 2 and 3). When the time-average tip-sample separation is in the range of 30−40 nm, however, the electrostatic force shows a weakly responsive gap state under small bias ranges across all the nanoflakes (Fig. 2b). A gap of 2.289 eV is observed on monolayer MoS_2_ which is consistent with its electronic bandgap^3,18^. We attribute this responsive gap to the bandgap of nanoflakes. As the sample is biased lower than −1.258 V (higher than +1.031 V), the Fermi level of the tip lies in the valance (conduction) band of monolayer MoS_2_ and the adequate DOS provides sufficient vacancy to accommodate charge carriers, and consequently, electric field builds up (Fig. 1g). Whereas, when the bias is set between −1.258 V and 1.031 V, the tip Fermi level is within the bandgap of monolayer MoS_2,_ and the zero DOS in the gap leads to a tiny quantum capacitance in the gap state at finite temperature as illustrated in Eq. (4). Therefore, the charge transfer cannot take place as in a classical way (Fig. 1f), and this is reflected in the electrostatic field on the tip. The noisy response of the EFM tip at the gap state could also be attributed to charging/discharging from the charge traps or adsorbates at the surfaces. When the time-average tip-sample separation reduces to under 30 nm, the curve starts to be chaotic, and this may be related to the fact that the repulsive van der Waals force dominates at such a short distance where the transient peak position of the EFM tip could be within a nanometre away from the samples.

**Fig. 3:**
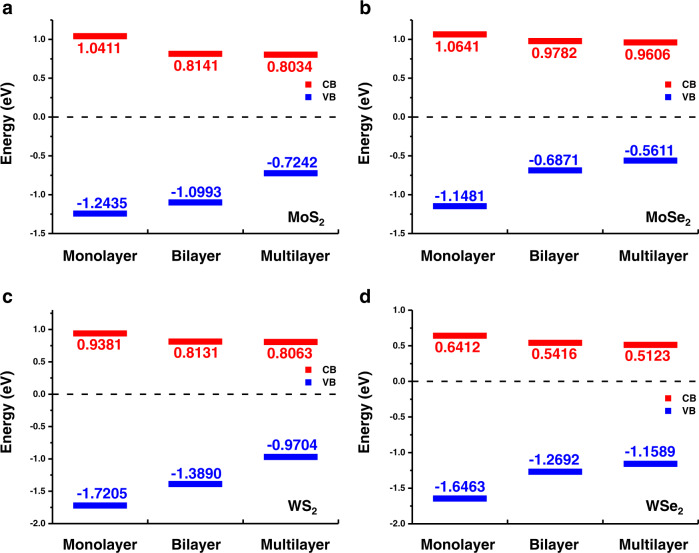
LEFM measurement results of several TMDC samples. Band edges of **a** MoS_2_, **b** MoSe_2_, **c** WS_2_, and **d** WSe_2_ revealed by the LEFM technique. The conduction band (CB) edges are marked in red and the valance band (VB) edges are in blue.

We demonstrate this technique in probing the band edges of various 2D semiconductors and heterostructures. Figure 3 depicts the representative band edges of monolayer, bilayer, and multilayer (with thickness around 20−40 nm) of these four TMDCs (MoS_2_, MoSe_2_, WS_2_, and WSe_2_) on ITO glasses which are characterized by this technique at ambient conditions. The experimental results are summarised in Table 1 and Supplementary Table 1. All the results are well consistent with the experimental reports with other techniques which are also listed in Table 1.

**Table 1:** The measured electronic band gap, the optical band gap (direct/indirect for bilayer sample) by photoluminescence spectroscopy, and the estimated exciton binding energy of four representative common TMDCs of different thicknesses

Unit (eV)		Electronic gap		Optical gap		Binding energy	
		Measured value	Reference value	Measured value	Reference value	Estimated value	Reference value
MoS_2_	monolayer	2.24 ± 0.04	2.11∼2.4^3,18,24–26^	1.891	1.81∼1.9^25–27^	0.352	0.09∼0.57^3,25,26,28,29^
	bilayer	1.91 ± 0.05	2.1^24^	1.88/1.60	1.59∼1.6^27,30^	0.311	
	multilayer	1.52 ± 0.03	1.29−1.75^24,31^				
MoSe_2_	monolayer	2.22 ± 0.02	1.9∼2.25^32–36^	1.575	1.56∼1.657^34,37,38^	0.641	0.59^34^
	bilayer	1.66 ± 0.04	1.56∼1.81^33,34^	1.57/1.52	1.55^34,37,38^	0.142	0.21^34^
	multilayer	1.51 ± 0.04	1.32^33^				
WS_2_	monolayer	2.63 ± 0.05	2.14∼2.73^18,26,39–41^	2.021	2.02∼2.09^40,41^	0.611	0.32∼0.71^26,28,40–42^
	bilayer	2.21 ± 0.03	1.82∼2.1^39,43^	1.99/1.76	1.68∼1.73^40,44^	0.45	
	multilayer	1.77 ± 0.04	1.4∼2.1^40,45^				
WSe_2_	monolayer	2.27 ± 0.06	1.75∼2.39^25,34^	1.647	1.61∼1.735^25,34,38,46^	0.618	0.14∼0.655^25,34,46,47^
	bilayer	1.81 ± 0.05	1.83^34^	1.64/1.43	1.54∼1.605^34,38^	0.377	0.23^34^
	multilayer	1.65 ± 0.03					

The related formerly reported results are listed for comparison.

It is noted that the bandgap probed by this technique reflects the electronic bandgap. The exciton binding energy could be estimated by the energy difference between the electronic gap and the optical gap which could be measured by photoluminescence (PL) spectroscopy. Our results are also summarized in Table 1 and the details could be found in Supplementary Figs. 2–5. The exciton binding energies we extracted are consistent with the former reports.

### LEFM measurement on TMDC heterostructures

**Fig. 4:**
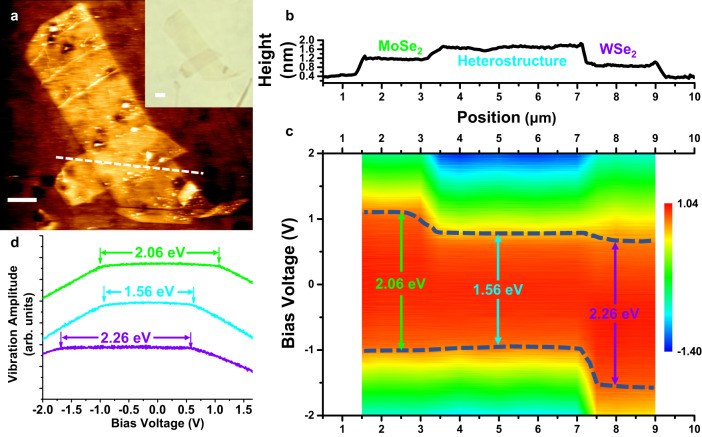
LEFM measurement on van der Waals heterostructures. **a** AFM topography mapping of the MoSe_2_/WSe_2_ van der Waals heterostructure. Inset is the optical image of the same sample (scale bars: 2 μm). **b** The height profile along the white dashed line in (**a**). **c** The band edge profile of the heterostructure along the white dashed line. The band gaps at the corresponding positions in (**b**) are marked by the color-corresponding arrows, and the band edges are highlighted by the dashed blue line. **d** The representative vibration amplitude vs. external bias curves obtained on the spots marked by the color-corresponding arrows in (**c**). The band edges are labeled by the arrows.

This technique provides a versatile tool to probe the band edge profile with a nanometre resolution. Figure 4 exhibits this technique in characterizing band alignment in a van der Waals heterostructure of monolayer MoSe_2_/WSe_2_. The band edge profile could be mapped at the ambient condition as shown in Fig. 4c. A clear type II bandgap alignment is identified in the area of the heterostructure, and this result is consistent with other experiment reports elsewhere^19–22^.

In summary, we demonstrate an extended EFM technique for the energy gap characterization on various 2D semiconductors and van der Waals heterostructures with nanometre resolution at ambient conditions.

## Methods

### Sample preparation

The monolayer and few-layer TMDC samples are cleaved from single crystals via the mechanical exfoliation method and dry-transferred to ITO glasses. For van der Waal heterostructures, monolayer samples are exfoliated onto a silicon substrate and the heterostructures are fabricated by the hot pick-up technique^23^ and eventually transferred onto heavily doped silicon carbide substrates.

### LEFM scanning

We move the EFM tip (BudgetSensors, Tap190E-G, k = 48 N/m, cantilever length = 225 μm) on top of the area of interest in non-contact working mode, lift the tip by tens of nanometres, and disable the z-feedback controller to maintain a constant tip-sample distance and then scan the external bias. The scanning bias is in the range of −2 V to 2 V. For 2D TMDC samples, the lift distance is in the range of 32−42 nm. The topography image shown in Fig. 4a was obtained with the AFM by Bruker (NanoWizard ULTRA Speed 2). The rest of the AFM/EFM/LEFM measurements were conducted with the LensAFM by Nanosurf.

### Photoluminescence measurement

Photoluminescence measurement is conducted with a homemade confocal PL spectrometer with a 532 nm continuous wave laser. The laser spot on the samples is ∼2 μm and the intensity was around 2 W/cm^2^.

## Supplementary information

Supplementary Information

## Data Availability

Relevant data supporting the key findings of this study are available within the article and the Supplementary Information file. All raw data generated during the current study are available from the corresponding authors upon request.
