# Supplementary material for: Electronic gap characterization at mesoscopic scale via scanning probe microscopy under ambient conditions
Source: Nat Commun. 2022 Aug 8;13:4648. doi: 10.1038/s41467-022-32439-1 (PMC9359982; doi:10.1038/s41467-022-32439-1)
Supplement: Supplementary file 1 — Supplementary Information [file 41467_2022_32439_MOESM1_ESM.pdf]

Supplementary Information for

Electronic gap characterization at mesoscopic scale via scanning probe microscopy under ambient conditions

Dian Li,[1,2] Xiong Wang,[1] Xiaoyong Mo,[3] Edmund C. M. Tse,[3] Xiaodong Cui[1]*

Affiliations:

[1]Department of Physics, Guangdong-Hong Kong Joint laboratory of Quantum Matter, University of Hong Kong, Pokfulam Road, Hong Kong SAR, P. R. China
[2]Department of Physics, Shantou University, Shantou, 515063, Guangdong, P. R. China
[3]Department of Chemistry, HKU-CAS Joint Laboratory on New Materials, University of Hong Kong, Pokfulam Road, Hong Kong SAR, P. R. China
*e-mail: xdcui@hku.hk

Supplementary Note 1. A simple model to estimate the charge distribution on the sample contributes to the electric field at the EFM tip.

We could estimate the charge distribution by an over-simplified model of a point charge on top of an infinite conductive plane with the separation d, as the schematic diagram shown below.

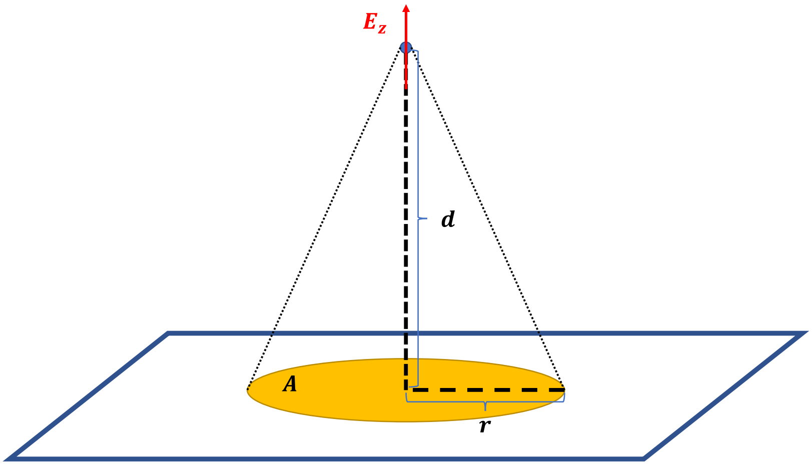

The electric field induced by the sample plane with an area of $A = \pi r^2$ that be felt by the tip is

$$E_z = \int_0^r \frac{qd}{4\pi\epsilon_0(r^2+d^2)^{\frac{3}{2}}} \cdot 2\pi r \cdot dr = -\frac{qd}{4\pi\epsilon_0(r^2+d^2)^{\frac{1}{2}}}\Bigg|_0^r$$

The localization coefficient η can be defined as the proportion that the area A influenced on the tip compared to the whole plane, namely,

$$\eta = \frac{E_z|_0^r}{E_z|_0^\infty} = \frac{\frac{1}{d} - \frac{1}{\sqrt{r^2+d^2}}}{\frac{1}{d}} = 1 - \frac{d}{\sqrt{r^2+z^2}} = 1 - \frac{1}{\sqrt{1+\left(\frac{r}{d}\right)^2}}$$

If we set the η to be 0.9, we can have

$$\frac{1}{\sqrt{1+\left(\frac{r}{d}\right)^2}} = 0.1 \Rightarrow r \sim 10d$$

That means for a point charge on top of an infinite conductive plane, 90% of the electrostatic interaction is localized at the area with the radius r is about ten times the separation d.

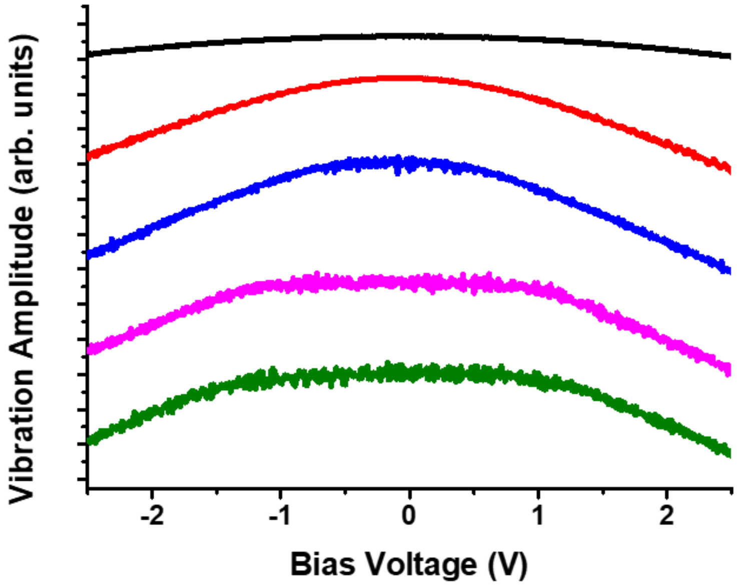

Supplementary Figure 1. The vibration amplitude of the EFM tip under various tip-sample distances. The vibration amplitude *vs.* external bias curves under different time-average tip-sample separation from 49 nm to 33 nm on monolayer MoS_2.

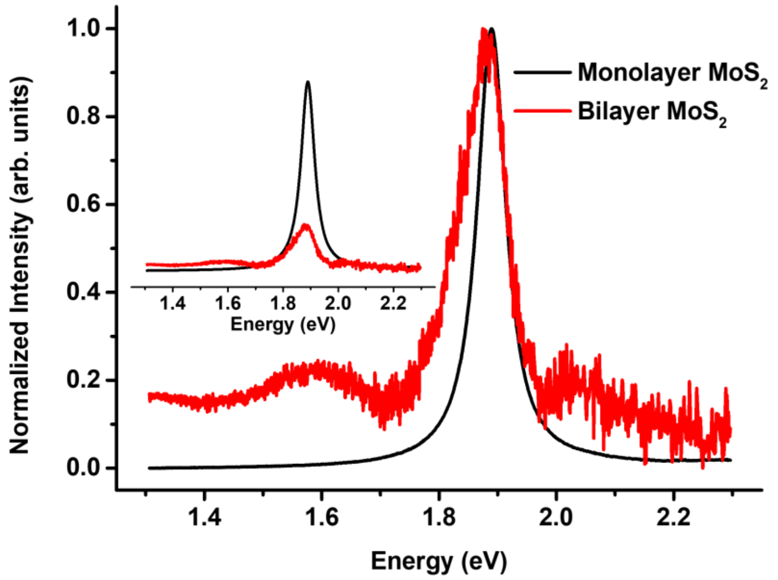

Supplementary Figure 2. PL characterizations on MoS_2. The PL signals of the monolayer (black) and bilayer (red) MoS_2 sample in normalized scale. The inset is the PL data with the same excitation power and exposure time.

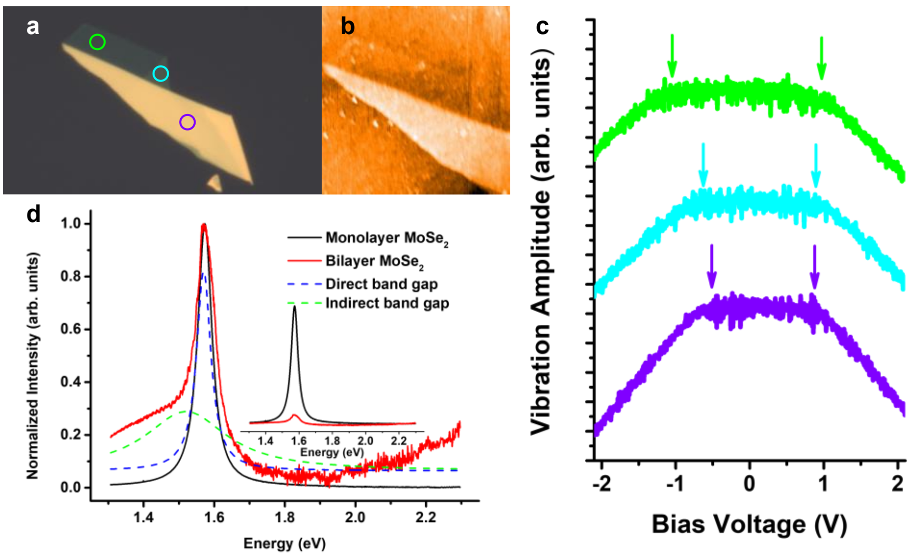

Supplementary Figure 3. LEFM and PL characterizations on $MoSe_2$ (a) The optical image and (b) the AFM topography mapping of the $MoSe_2$ sample. The areas of monolayer, bilayer, and multilayer (layer number larger than 20) are circled in green, blue, and violet, respectively. (c) The representative vibration amplitude *vs.* external bias curves under the LEFM working mode on the corresponding areas in (a) are marked as the same colour. The arrows indicate the edges. (d) the PL signals of the monolayer (black) and bilayer (red) $MoSe_2$ sample in normalized scale. The inset is the PL intensity data with the same excitation power and exposure time.

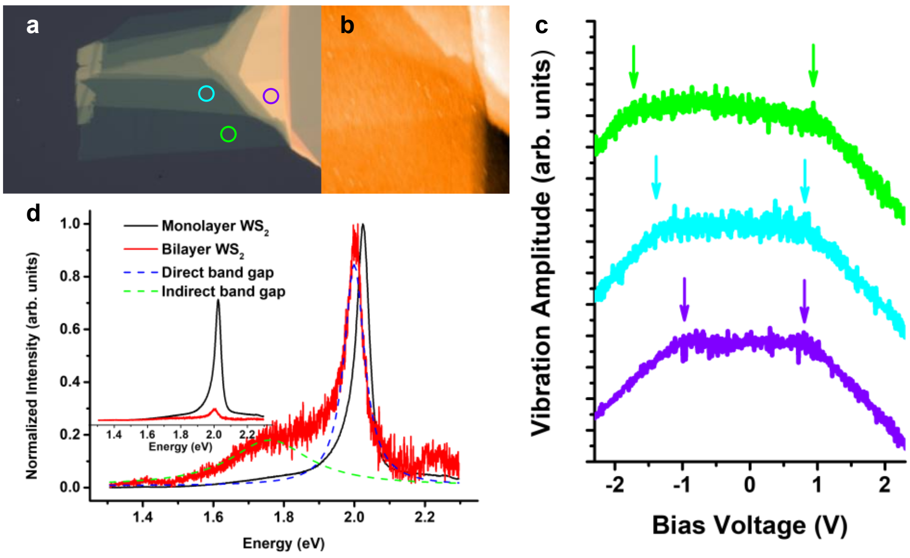

Supplementary Figure 4. LEFM and PL characterizations on WS_2 (a) The optical image and (b) the AFM topography mapping of the WS_2 sample. The areas of monolayer, bilayer, and multilayer (layer number larger than 20) are circled in green, blue, and violet, respectively. (c) The representative vibration amplitude *vs.* external bias curves under the LEFM working mode on the corresponding areas in (a) are marked as the same colour. The arrows indicate the edges. (d) the PL signals of the monolayer (black) and bilayer (red) WS_2 sample in normalized scale. The inset is the PL intensity data with the same excitation power and exposure time.

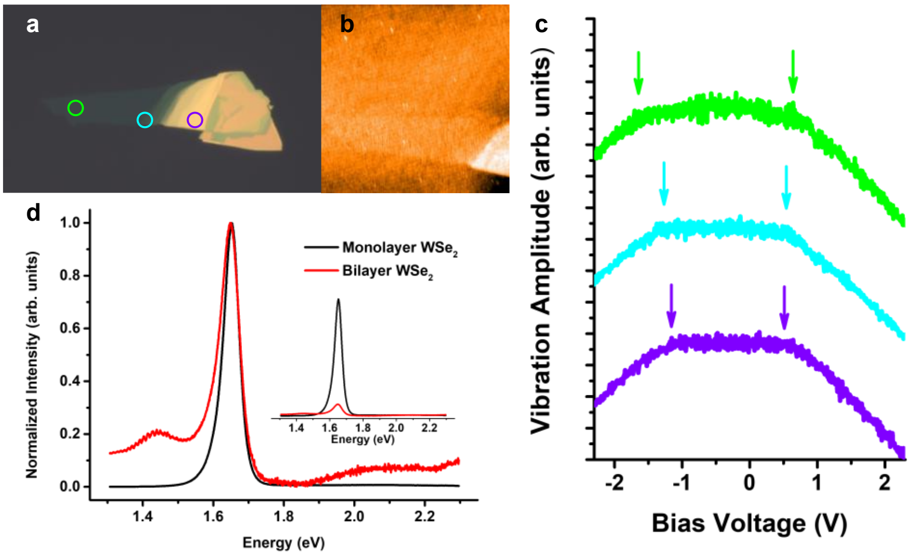

Supplementary Figure 5. LEFM and PL characterizations on WSe_2 (a) The optical image and (b) the AFM topography mapping of the WSe_2 sample. The areas of monolayer, bilayer, and multilayer (layer number larger than 20) are circled in green, blue, and violet, respectively. (c) The representative vibration amplitude *vs.* external bias curves under the LEFM working mode on the corresponding areas in (a) are marked as the same colour. The arrows indicate the edges. (d) the PL signals of the monolayer (black) and bilayer (red) WSe_2 sample in normalized scale. The inset is the PL intensity data with the same excitation power and exposure time.

Supplementary Table 1. The statistical results of the bias voltages of the corresponding platform edges and the measured electronic band gap with 9 measurements in each area.

		left edge (V)	right edge (V)	electronic gap (eV)
MoS_2	monolayer	-1.227±0.031	1.016±0.04	2.243±0.045
	bilayer	-1.100±0.042	0.811±0.018	1.911±0.048
	multilayer	-0.713±0.026	0.804±0.011	1.517±0.029
$MoSe_2$	monolayer	-1.140±0.03	1.075±0.033	2.216±0.019
	bilayer	-0.686±0.021	0.976±0.041	1.662±0.043
	multilayer	-0.555±0.025	0.957±0.034	1.512±0.037
WS_2	monolayer	-1.704±0.039	0.929±0.022	2.632±0.046
	bilayer	-1.393±0.018	0.815±0.021	2.309±0.033
	multilayer	-0.974±0.035	0.798±0.023	1.773±0.041
WSe_2	monolayer	-1.645±0.054	0.621±0.048	2.265±0.063
	bilayer	-1.254±0.046	0.552±0.01	1.807±0.046
	multilayer	-1.143±0.026	0.502±0.009	1.645±0.031
